# The Chicken or the Egg? Understanding the Temporal Relationship Between Severe Mental Illness and Neurological Conditions in a UK Primary Care Cohort

**DOI:** 10.1192/bjo.2024.79

**Published:** 2024-08-01

**Authors:** Ella Burchill, Jonathan Rogers, David Osborn, Joseph Hayes, Naomi Launders

**Affiliations:** Division of Psychiatry, University College London, London, United Kingdom

## Abstract

**Aims:**

A significantly higher prevalence of neurological conditions has been found both before and after a diagnosis of schizophrenia, bipolar disorder and other psychotic illnesses compared with the general population.

We aimed to understand the cumulative prevalence of 16 neurological conditions in people with severe mental illness (SMI) from 5 years before to 5 years after their SMI diagnosis. We hypothesised that individual neurological conditions would have differential temporal relationships relative to SMI diagnosis.

**Methods:**

In a longitudinal matched study, we identified a cohort of patients aged 18–100 years from Jan 1, 2000, and Dec 31, 2018, from the UK Clinical Practice Research Datalink (CPRD). Neurological conditions were classified using ICD–11 criteria into umbrella clusters of disease. Outcome of interest was a diagnosis of SMI. Each SMI patient was matched 1:4 to patients without SMI in the CPRD cohort, matching for sex, 5-year age band, primary care practice and year of practice registration. The cumulative prevalence of 16 neurological conditions was recorded cross-sectionally at 5, 3, 1 years prior to SMI diagnosis, at SMI diagnosis (index), and 1, 3 and 5 years after SMI diagnosis. Logistic regression modelling aided comparison of differential prevalence of neurological conditions, adjusting for sociodemographic variables, and with further adjustment for body mass index, smoking, alcohol and non-prescription drug use. Multiple imputation was applied in cases of missing data.

**Results:**

We identified 68,789 patients with SMI, matched to 274,827 controls. The median age was 40.9 years, 49.05% of the overall cohort were female (33,783 SMI patients, 134,740 controls), and the majority were of White ethnicity (35,228, 51.2% SMI patients, 125,518, 45.7% controls). The most prevalent neurological conditions across seven timepoints were cerebral palsy, cerebrovascular disease, dementia, epilepsy, multiple sclerosis, paralysis and Parkinson's disease. Conditions with the highest fully adjusted odds ratios (ORs) for SMI diagnosis were dementia 3 years after SMI diagnosis (5.32, 95% CI 4.95–5.71) and Parkinson's disease 5 years after SMI diagnosis (4.26, 95% CI 3.68–4.94).

**Conclusion:**

All 16 neurological conditions have higher prevalence in the SMI cohort compared with controls, with different prevalence patterns observed over the 10-year study period. A consistently lower OR for schizophrenia compared with other SMI warrants further exploration, as neurological conditions risk being under-recorded.

A greater understanding of the temporal relationship between SMI and neurological conditions may help promote earlier diagnosis, increased screening and better holistic management of both conditions.

